# Supplementary material for: Ecological Interactions among Thrips, Soybean Plants, and Soybean Vein Necrosis Virus in Pennsylvania, USA
Source: Viruses. 2023 Aug 18;15(8):1766. doi: 10.3390/v15081766 (PMC10458877; doi:10.3390/v15081766)
Supplement: Supplementary file 1 [file viruses-15-01766-s001.zip › viruses-2540061-supplementary.pdf]

Table S1. Quantification of virus titers for the establishment of protocol for SVNV inoculation through qRT PCR

Treatment	Gene	CT Value
Infected Plant	NP gene	37.10885
Infected Plant	NP gene	36.60115
Healthy Plant	NP gene	NaN
Healthy Plant	NP gene	NaN
Water	NP gene	NaN
Water	NP gene	NaN
Mechanical Inoculation	NP gene	NaN
Mechanical Inoculation	NP gene	NaN
Syringe Inoculation	NP gene	37.18007
Syringe Inoculation	NP gene	35.90067
Thrips 5 (Transmission)	NP gene	38.22273
Thrips 5 (Transmission)	NP gene	38.11723
Thrips 10 (Transmission)	NP gene	36.75315
Thrips 10 (Transmission)	NP gene	35.85314
Thrips 15 (Transmission)	NP gene	36.19639
Thrips 15 (Transmission)	NP gene	38.25706
Infected plant	SE gene	30.02398
Infected plant	SE gene	29.83493
Healthy plant	SE gene	39.57612
Healthy plant	SE gene	39.57612
Water	SE gene	NaN
Water	SE gene	NaN
Mechanical Inoculation	SE gene	32.19139
Mechanical Inoculation	SE gene	31.9264
Syringe Inoculation	SE gene	32.64924
Syringe Inoculation	SE gene	32.69367
Thrips 5 (Transmission)	NV gene	27.00357
Thrips 5 (Transmission)	NV gene	27.11734
Thrips 10 (Transmission)	NV gene	28.8004
Thrips 10 (Transmission)	NV gene	28.48253
Thrips 15 (Transmission)	Nvgene	34.45782

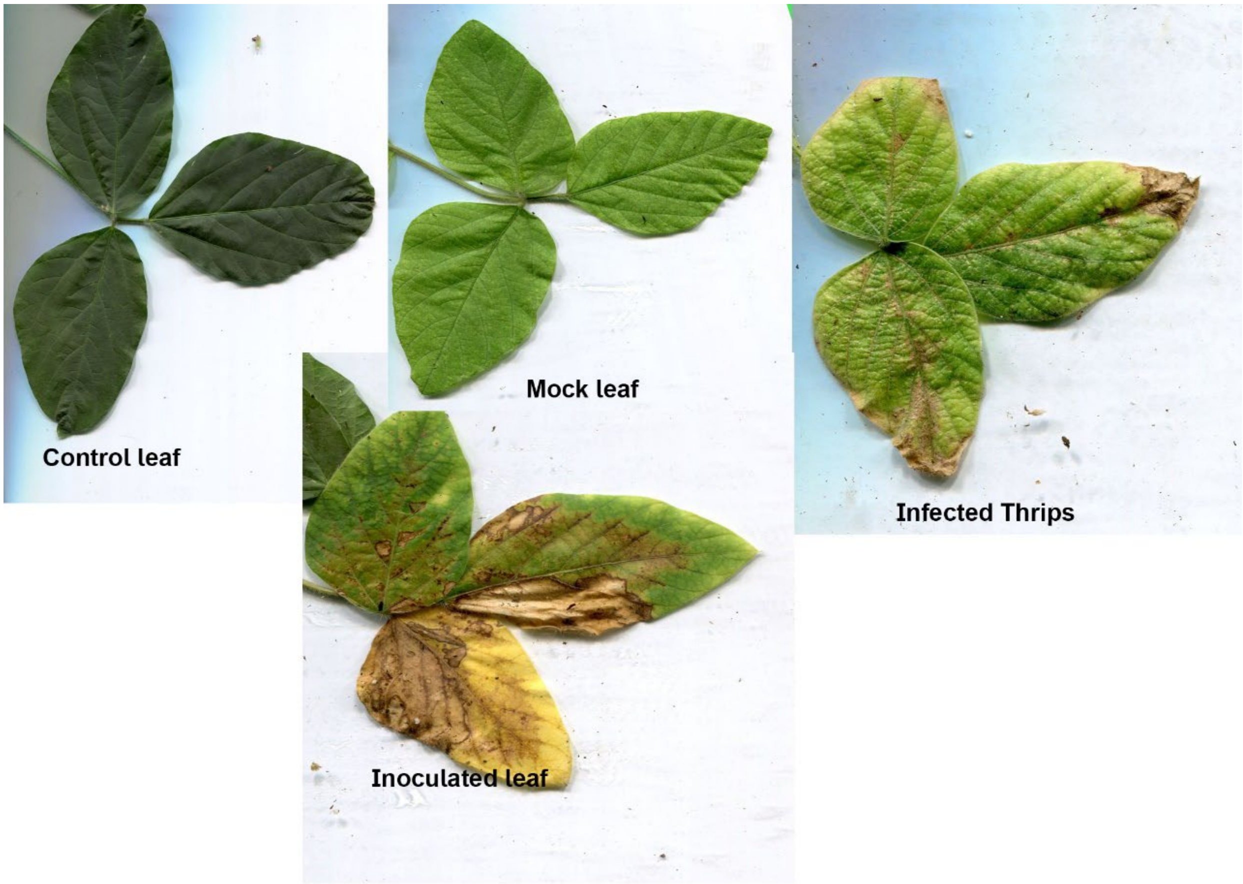

Figure S1. Plant leaves across the different treatments.

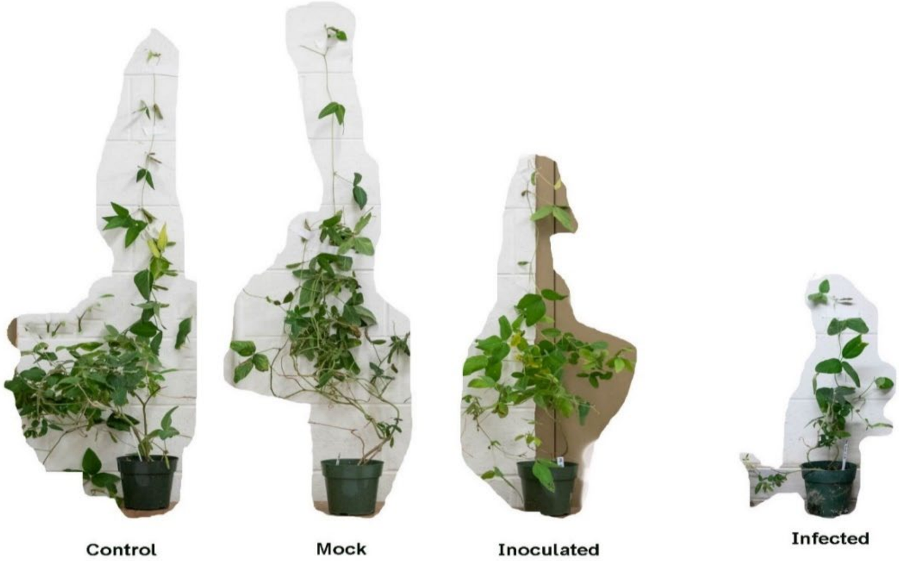

Figure S2. Effect of soybean vein necrosis virus (SVNV) on general plant health.

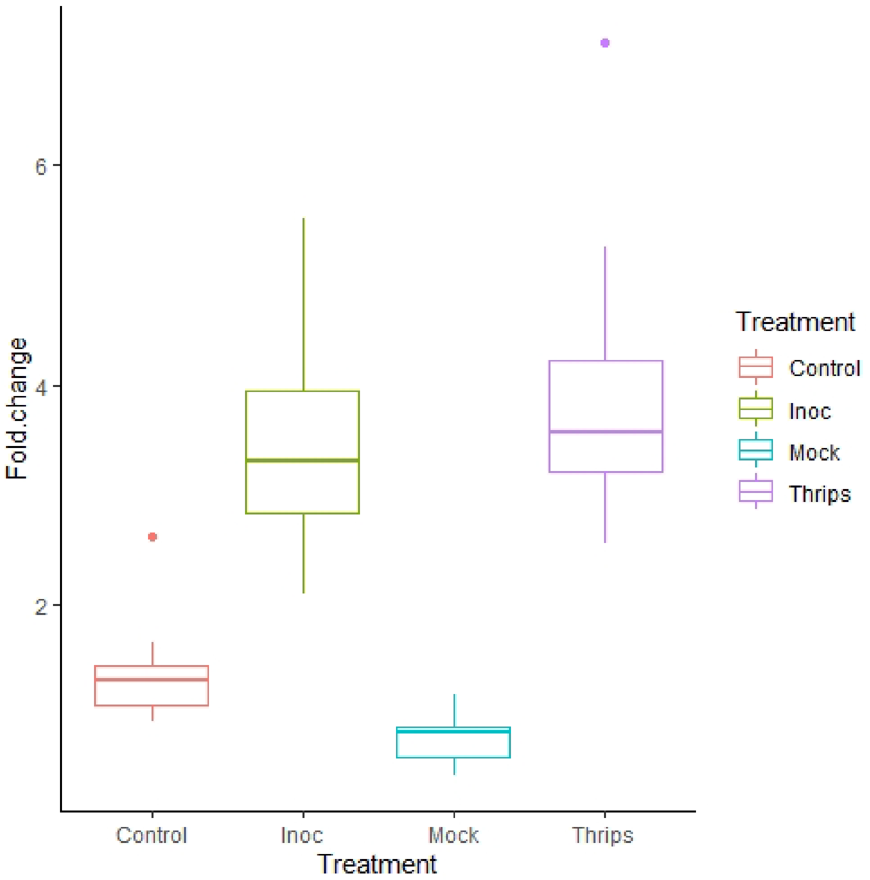

Figure S3. After one month of inoculation, plants of different treatments were checked with ELISA (Agdia, USA) for virus presence and compared with buffer PBST. OD values taken through the ELISA plate reader at 405 nm were used to compare the virus titers across the treatments. The plants that had ELISA values greater that 3x the control or the buffer treatment were considered positive for soybean vein necrosis virus (SVNV). Here Inoc= mechanically inoculated plants. Thrips= SVNV + Thrips infected plants, control = healthy uninfected plants, mock= plants syringe included with buffer only.
